# Anticancer Activity of *Cordia dichotoma* against a Panel of Human Cancer Cell Lines and Their Phytochemical Profiling via HPLC and GCMS

**DOI:** 10.3390/molecules27072185

**Published:** 2022-03-28

**Authors:** Shilpa Raina, Vikas Sharma, Zahid Nabi Sheikh, Navneet Kour, Shashank K. Singh, Ali Zari, Talal A. Zari, Hesham F. Alharby, Khalid Rehman Hakeem

**Affiliations:** 1 Division of Biochemistry, Faculty of Basic Sciences, Sher-e-Kashmir University of Agricultural Sciences and Technology of Jammu, Main Campus Chatha, Jammu 180009, India; shilparaina70@gmail.com (S.R.); sheikhzahid375@gmail.com (Z.N.S.); kour.navneet13@gmail.com (N.K.); 2 Cancer Pharmacology Division, CSIR-Indian Institute of Integrative Medicine, Jammu 180001, India; sksingh@iiim.res.in; 3 Department of Biological Sciences, Faculty of Science, King Abdulaziz University, Jeddah 21589, Saudi Arabia; azari@kau.edu.sa (A.Z.); talzari@yahoo.com (T.A.Z.); halharby@kau.edu.sa (H.F.A.); 4 Princess Dr Najla Bint Saud Al-Saud Center for Excellence Research in Biotechnology, King Abdulaziz University, Jeddah 21589, Saudi Arabia; 5 Department of Public Health, Daffodil International University, Dhaka 1341, Bangladesh

**Keywords:** *C. dichotoma*, anticancer, apoptosis, ROS, HPLC, GCMS

## Abstract

The current study was conducted to examine the in vitro anticancer potential of *Cordia dichotoma* (bark, leaves, pulp and seed). The plant material was collected from UT of J&K and methodical bioassays were carried out on ten human cancer cell lines (Michigan Cancer Foundation-7 (MCF-7), M.D. Anderson-Metastatic Breast (MDA-MB-231), Neuroblastoma-2a (N2A), SH-SY5Y, U-251, HCT-116, SW-620, A-549, MIA PaCa-2, Panc-1) from five different origins (breast, CNS, colon, lung, pancreas) respectively. Methanolic extracts were produced and fractions were then obtained from the extracts and evaluated for cytotoxicity. Mechanistic assays, HPLC, and GCMS profiling were performed on the highest active fraction. The Sulforhodamine B (SRB) assay determined the in vitro cytotoxicity. The findings revealed that the bark portion had in vitro cytotoxicity against the A-549 human lung cancer cell line. To our knowledge, this is the first study to show that the plant’s bark has anticancer properties and induced chromatin condensation, confirmed cell death via ROS generation, and significantly decreased colony formation in A-549 cell line from lung origin in a dose-dependent manner. Furthermore, HPLC and GCMS investigations indicated the presence of a number of bioactive molecules such as gallic acid (144,969.86) uV*sec, caffeic acid (104.26) uV*sec, ferulic acid (472.87) uV*sec, vanillic acid (13,775.39) uV*sec, palmitic acid (18.34%), cis vaccenic acid (28.81%), etc. and one of the compounds was reported for the first time from the bark. As a result of its promising efficacy, it may become an essential cancer chemopreventive or chemotherapeutic medication for patients with lung carcinoma.

## 1. Introduction

Cancer is one of the most persistent diseases in humans and there is a lot of scientific and economic attention in finding novel anticancer medicines in natural sources, including terrestrial and aquatic plants. Natural compounds originating from plants play an vital part in the expansion and marketing of pharmaceuticals [1,2]. The revelation of drugs from natural sources is associated with their ethno-pharmacological utilization and the heft of plant-inferred medicines are likewise connected to their conventional restorative use from quite some time ago [3,4]. Terpenoids, phenolics, flavonoids, and alkaloids are the principal types of plant-derived compounds with therapeutic effects [2]. These substances act on a variety of cellular components, causing alterations in the cell’s regular metabolism and growth. Although the majority of anticancer drugs isolated from different parts of the plants used in chemotherapy such as Vincristine and Vinblastime from *Catharanthus roseus*, Taxol from *Taxus brevifolia*, and camptothecin from *Camptotheca acuminata* [5,6,7,8] are recommended, which alter the basic biological processes [9,10] and impede nucleic acid synthesis, however the mechanisms of action differ significantly. A number of mechanisms [11,12], including mitochondrial pathways [13], can cause apoptosis. It is generally known that reactive oxygen species (ROS) may cause intracellular protein modification, lipid peroxidation, mitochondrial malfunction, and finally apoptosis [14,15]. ROS are important in cell signaling and the control of the primary apoptosis pathways mediated by mitochondria, death receptors, and the endoplasmic reticulum. Other controlled mechanisms of cell survival and death, such as autophagy and necroptosis, are also influenced by ROS [16]. The involvement of ROS in the intricate interaction and crosstalk between these diverse signaling pathways, in particular, will be investigated extensively in the coming years.

*Cordia dichotoma* (lasoda) is a member of the Boraginaceae family with around 2740 species divided into 148 genera and has been historically used to treat a number of human ailments in India. *Cordia* is a significant and prominent genus within this family. It is found in the Sub-Himalayan and outlying slopes of the Himalayas. It grows in a wide range of forests, from the Rajasthan to the India’s Western Ghats [17]. The species is common in the Philippines and can be found in thickets. The species is propagated by seeds. Leaves are elliptic-lanceolate to long ovate, simple, whole, and slightly dentate with a round and cordate base. The flowers have small bisexual stalks and vary in color from white to pink. The fruits with a sticky flesh mass are edible, immature fruits and are pickled/used as vegetables and the whole plant of *C. dichotoma* is edible/used as food [18]. According to ethnopharmacological studies, the plant has antimicrobial, antifungal [19], wound healing [20], antiinflammatory [21], and antibiofilm [22] properties.

The intention of this study was to determine the anticancer potential of different parts of *C. dichotoma* against ten human cancer cell lines from five different origins such as breast (MCF-7, MDA-MB-231), CNS (N2A, SH-SY5Y, U-251), colon (HCT-116, SW-620), lung (A-549), and pancreas (MIA PaCa-2, Panc-1), as well as to assess the potential for cellular oxidative stress at intracellular ROS levels, nuclear morphology, wound healing, and mitochondrial permeability. Furthermore, we investigated the bioactive compound responsible for the activity through GCMS and HPLC profiling.

## 2. Results

### 2.1. Screening for Cytotoxic Activity

**Figure 1:**
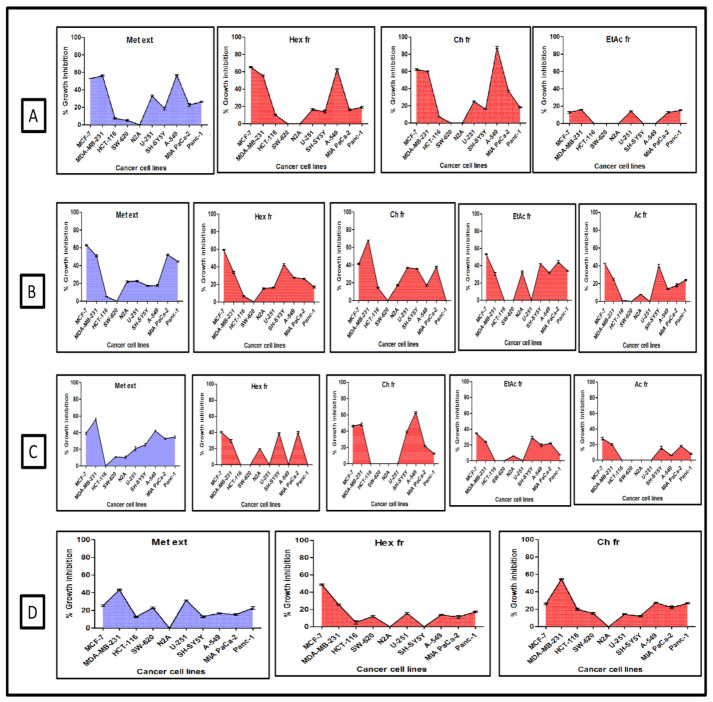
Graphs represent in vitro cytotoxic potential of different parts of *C. dichotoma* Bark (**A**), Leaves (**B**), Pulp (**C**), and Seed (**D**) extracts and fractions at concentration 100 μg/mL. Values are the mean ± SD (*n* = 3). Met ext (Methanolic extract), Hex fr (Hexane fraction), Ch fr (Chloroform fraction), EtAc fr (Ethyl acetate fraction), and Ac fr (Acetone fraction).

The SRB test was used to examine the cytotoxic impact of extracts and fractions from *C. dichotoma* bark, leaves, pulp, and seed on the development of various human cancer cell lines and the criteria for activity was a minimum of 70%. Figure 1 depicts the cell inhibitory activity of methanol extract and subsequent fractions (hexane, chloroform, ethyl acetate, and acetone).

In the preliminary investigation of methanolic extract obtained from the bark of *Cordia dichotoma*, against ten human cancer cell lines (MCF-7, MDA-MB-231, N2A, SH-SY5Y, U-251, HCT-116, SW-620, A-549, MIA PaCa-2, Panc-1) from five different origins (breast, CNS, colon, lung, pancreas) respectively, the results showed growth inhibition in the range of 0–57%. However, the chloroform fraction obtained from the abovementioned extract showed remarkable results as a growth inhibition of 88% was found against the A-549 cell line from lung tissue, whereas the same fraction inhibited the human cancer cell lines used in the present study in the range of 0–62%. While evaluating the cytotoxic potential of other fractions, the growth inhibition in case of n-hexane fraction was observed in the range of 0–65%, and for ethyl acetate fraction the growth inhibition range was 0–16% as shown in (Figure 1A). The acetone fraction was completely considered as inactive as growth inhibition was found as 0%.

The lasoda leaf extract (methanolic) and fractions were evaluated at the concentration of 100 µg/mL against ten human cancer cell lines used in the present study. The growth inhibition by extract was found in the range of 0–63%, whereas in case of fractions, the growth inhibition was in the range of 0–59% in n-hexane, 0–68% in chloroform, 0–53% in ethyl acetate, and 0–43% in acetone fraction as depicted in Figure 1B.

The pulp part of the lasoda fruit was assessed at the same concentration against ten human cancer cell lines used in the present investigation. The overall growth inhibition was seen in the range of 0–56% in methanolic extract, 0–40% in n-hexane fraction, 0–63% in chloroform fraction, 0–34% in ethyl acetate fraction, and 0–27% in acetone fraction as shown in Figure 1C. So, the pulp part of the lasoda fruit was considered as non-significant. The observations produced by the methanolic extract and subsequent fractions from the seed part of *C. dichotoma* are shown in Figure 1D and the data represented that the growth inhibition by the extract was observed in the range of 0–43%. The growth inhibition in case fractions, namely *n*-hexane and chloroform, was observed in the range of 0–48% and 0–54% respectively, which is considered non-significant. The ethyl acetate and acetone fractions were considered purely inactive as growth inhibition was found as 0%.

The chloroform fraction was further evaluated at lower concentrations (50, 40, 30, 20, 10 µg/mL) against the A-549 (lung) cancer cell line. Table 1 depicts the IC_50_ value of chloroform fraction and standard drug.

### 2.2. Mechanistic Assays

**Figure 2:**
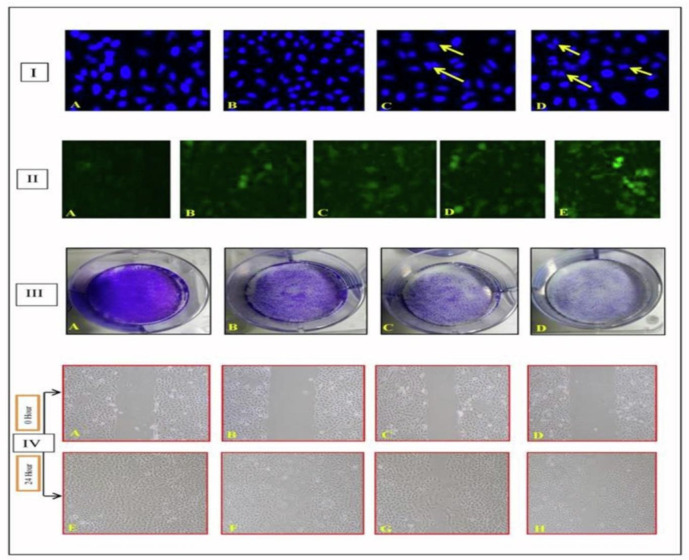
Various mechanistic assays performed on human lung cancer cell line A-549. **I.** Nuclear morphological changes as indicated by arrow using DAPI staining with chloroform fraction of bark treatment. (**A**) Control (**B**) 50 µg/mL (**C**) 75 µg/mL and (**D**) 100 µg/mL **II.** Effect of chloroform fraction on A-549 in the production of reactive oxygen species evaluated by oxidation of DCFDA by hydrogen peroxide through fluoroscence microscopy at 24 h posttreatment. (**A**) Control (**B**) H_2_O_2_ 0.5% (**C**) 50 µg/mL (**D**) 75 µg/mL (**E**) 100 µg/mL **III.** In vitro colony formation assay of bark chloroform fraction (**A**) Control (**B**) 50 µg/mL (**C**) 75 µg/mL and (**D**) 100 µg/mL **IV.** In vitro wound healing assay chloroform fraction. 0 h (**A**) Control (**B**) 50 µg/mL (**C**) 75 µg/mL and (**D**) 100 µg/mL. After 24 h (**E**) Control (**F**) 50 µg/mL (**G**) 75 µg/mL and (**H**) 100 µg/mL.

The chloroform fraction displayed a potent in vitro cytotoxic effect against human lung cancer cell line (A-549) with maximum growth inhibition and lower IC_50_ values. So, the fraction was chosen for several mechanistic experiments against the A-549 human lung cancer cell line as shown in Figure 2.

#### 2.2.1. Chloroform Fraction Triggered Chromatin Condensation

The fluorescent dye DAPI (4,6-diamidino-2-phenylindole) binds to the A-T region of DNA’s minor groove. The activation of apoptosis results in morphological changes such as chromatin condensation, cell shrinkage, nuclear distortion, and the development of apoptotic bodies and their elimination by neighboring cells. After being treated with different concentrations of chloroform fraction, namely 50, 75, and 100 µg/mL, A-549 cells were stained with DAPI dye and studied under a fluorescence microscope. Untreated cells exhibited a constant appearance of blue nuclei, indicating that they were healthy. The chloroform fraction-treated cells, on the other hand, demonstrated dose-dependent chromatin condensation of cell nuclei as shown in Figure 2I. After a 24-h incubation period, the size and shape of cell nuclei in drug-treated cells seemed to change. The fraction induced DNA fragmentation as well as a decrease in the number of cell nuclei in a concentration-dependent manner, according to the findings.

#### 2.2.2. ROS Production by the Chloroform Fraction Causes Cell Death

DCFDA (2,7-dichlorofluorescein diacetate), a non-fluorescent dye that becomes green in the presence of ROS, was used to detect its presence. It is a hydrogen peroxide-detecting probe used to assess hydrogen peroxide levels in intact cells. It easily penetrates across the plasma membrane and oxidizes in the presence of free radicals to generate DCF (dichlorofluorescein), a green fluoroscent byproduct that is excited at 495 nm and emits at 520 nm. When treated with DCFDA dye, the positive control (0.5% H_2_O_2_) emitted green fluorescence, which was then compared with drug-treated cells. The results indicate a substantial dose-dependent increase in the fluorescence of drug-treated (chloroform fraction) cells, demonstrating that ROS generation is required for cell death as shown in Figure 2II.

#### 2.2.3. Effect of Fraction on Colonies Development

It is an in vitro cell existence test that is based on the ability of a solitary cell to become a colony. This test evaluates the ability of each cell in a population to divide forever and is used to determine the effectiveness of cytotoxic drugs. It assists in assessing if cells have retained the potential to generate a large number of progenies or have perished as a result of various pharmacological treatments. Before seeding in the appropriate dilution, A-549 cells were treated with chloroform fraction at concentrations of 50, 75, and 100 µg/mL. The chloroform fraction of bark inhibited colony development in A-549 cells in a dose-dependent manner, according to the findings. As indicated in Figure 2III, the colony formation of the control cells was higher than that of the drug-treated cells.

#### 2.2.4. Inhibition of Cell Migration by Chloroform Fraction

An in vitro wound healing assay was performed to evaluate the effect of chloroform fraction on the migration of A-549 cells. Following the drug treatment, images of cells moving into the scratched region were captured. The results indicated that wound closure was clearly evident in untreated cells after 24 h due to cell migration and cell proliferation, but cell migration was hindered in chloroform fraction treated cells with an increasing concentration of 100 µg/mL, where the scratch produced was clearly visible. As a result of the foregoing findings, the antiproliferative impact of fraction treatment resulted in impaired cell migration shown in Figure 2IV.

### 2.3. Identification of Anticancer Molecules

Motivated by the cytotoxicity findings and mechanistic assays, we tried to identify the positive molecules responsible for anticancer activity by suitable characterization investigations such as high-performance liquid chromatography (HPLC) and gas chromatography mass spectrometry (GCMS).

#### 2.3.1. High-Performance Liquid Chromatography

**Figure 3:**
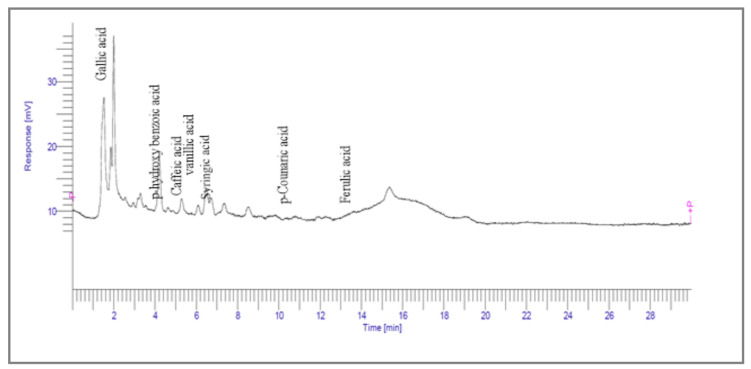
HPLC chromatogram of Chloroform fraction of bark of *C. dichotoma*.

HPLC analysis was used to detect certain key pharmaceutical substances. Peaks were identified by comparing the retention period of the chloroform fraction of *C. dichotoma* bark with those of the reference substances. The generated peaks were proportioned to each constituent as shown in Figure 3. 

Phenolics are one of nature’s most diverse chemical families. Phenolic compounds are phytochemicals present in almost all plant tissues. They are secondary metabolites produced by the shikimic acid and phenylpropanoid pathways, respectively. They have a variety of bioactive characteristics [23]. HPLC quantification of the fraction identified the presence of gallic acid, syringic acid, p coumarin acid, caffeic acid, p-hydroxy benzoic acid, ferulic acid, and vanillic acid as depicted in (Table 2). Gallic acid and vanillic acid constituted the highest content observed with the areas of 144,969.86 and 13,775.39 uV*sec respectively.

#### 2.3.2. Gas Chromatography Mass Spectroscopy Analysis

**Figure 4:**
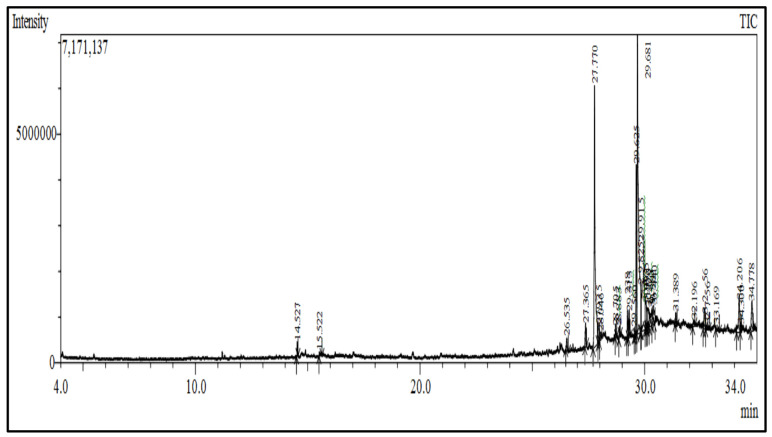
GCMS chromatogram of the chloroform fraction of bark of *C. dichotoma*.

Chloroform fraction is composed of organic molecules that are volatile in nature, primarily fatty acids. Twenty six compounds were identified by GCMS as shown in the spectrum in Figure 4.

The compounds found in GCMS are presented in Table 3. Among all compounds, the highest percentage compound was *cis*-vaccenic acid followed by palmitic acid, palmitoleic acid, Octadecadienoic acid, and soon. Furthermore, to the best of our knowledge, it is the first time that *cis*-vaccenic acids, already described in other plants, were reported in the lasoda bark.

## 3. Discussion

The burden of cancer rose to 18.1 million new cases and 9.6 million deaths in 2018 and with 36 different types, cancer mainly affects men in the form of colorectal, liver, lung, prostate, and stomach cancer and women in the form of breast, cervix, colorectal, lung, and thyroid cancer [24]. However, a projected 19.3 million new cancer cases and about 10.0 million cancer deaths occurred globally in 2020. With an anticipated 2.3 million new cases (11.7%), female breast cancer has surpassed lung cancer as the most often diagnosed malignancy, followed by lung (11.4%), colorectal (10.0%), prostate (7.3%), and stomach (5.6%). With a projected 1.8 million fatalities (18%), lung cancer remains the top cause of cancer mortality, followed by colorectal (9.4%), liver (8.3%), stomach (7.7%), and female breast (6.9%). Overall, the prevalence of both sexes was 2- to 3-fold greater in transitioned versus transitional nations, although death differed 2-fold for males and little for females [25], and treating cancer has become a whole new area of research. There are conventional as well as modern techniques applied against cancer and a variety of techniques like chemotherapy, radiation therapy, and surgery, which are used for treating the disease. However, all of them have some disadvantages [26] as the use of conventional chemicals bear side effects/toxicities **[27]**, but as the problem persists, new approaches are needed for the control of disease, especially because of the failure of conventional chemotherapeutic approaches. Therefore, there is a need for new strategies for the prevention/cure of cancer to control the death rate and plant derived components has become a very safe, non-toxic and easily available source as botanicals are believed to neutralize the effects of diseases in a body because of their numerous medicinal properties [1,2,3,4]. In the present investigation, we evaluated the in vitro cytotoxic potential of *Cordia dichotoma* bark, leaves, pulp, and seed, collected from the Union Territory of Jammu & Kashmir. The extracts (methanolic) and subsequent fractions (*n*-hexane, chloroform, ethyl acetate, acetone, methanol soluble) were screened against ten human cancer cell lines (MCF-7, MDA-MB-231, N2A, SH-SY5Y, U-251, HCT-116, SW-620, A-549, MIA PaCa-2, Panc-1,) originated from five different tissues (breast, CNS, colon, lung, pancreas) respectively and the anticancer activity was produced by only chloroform fraction of bark as it fetched the IC_50_ value 37.978 µg/mL. The fraction was further used for mechanistic assays and it induced chromatin condensation, confirmed cell death via ROS generation, and significantly decreased colony formation in a dose-dependent manner in the A-549 cell line from lung origin.

Furthermore, when compared with the work of others, Hussain et al. [28] observed 69.4 and 72.1 percent growth inhibition in methanolic extract of bark against MDA-MB-231 and MCF-7 respectively. The methanolic extract of *C. dichotoma* leaves was tested for anticancer activity against human prostate carcinoma cell line (PC3) and human cervical cancer cell line (HeLa), and the results indicate that the extract exhibited good anti-cancerous activity against HeLa [29]. According to some publications, the antitumor and anticancer actions of *C. dichotoma* fruit pulp extract may be attributed to its antioxidant ability due to its high level of secondary metabolites [30]. Healthy cells maintain a correct equilibrium of ROS levels, but stress situations cause ROS levels to overwhelm the antioxidant capacity on defense, resulting in an oxidative stress state. Transcription factors play a significant part in the regulation of cellular growth cycle, tumor survival and angiogenesis [31]. The methanolic extract and aqueous portion of *Cordia dichotoma* seeds are cytotoxic to human cervical epitheloid (HeLa) [32]. The SRB test was used to assess the cytotoxic activity of *Cordia myxa* fruit extract and its micro and nano capsulated forms in vitro using THLE2 and HepG-2 cell lines. The fruit extract was more cytotoxic (IC_50_: 70 g/mL) than the micro and nanocapsules (IC_50_: 100 g/mL). The reduction in viability of hepatocellular carcinoma cells (HepG-2) treated with extract compared with treated normal cells (THLE2) demonstrated that the fruit extract was selective against only malignant cells. The extract inhibited cell proliferation less effectively against the HepG2 cell line. Further, micro and nanocapsules showed a very low cytotoxicity [33]. In our current study, the growth inhibition of various extracts and fractions deviated somewhat from that described in the literature. This might be due to regional differences, solvent polarities or extraction methods. The data regarding in vitro cytotoxicity of *C. dichotoma* bark against A-549, is not available in literature, made us unable to compare our results.

Our study further investigates the bioactive compound which might be responsible for the activity through phytochemical profiling via HPLC and GCMS. We investigate seven phenolic standards such as gallic acid, syringic acid, *p*-coumarin acid, caffeic acid, *p*-hydroxy benzoic acid, ferulic acid and vanillic acid. Ferulic acid, caffeic acid, gallic acid, and syringic acid have already been explored for their anticancer potential [34,35,36,37]. By regulating the JAK/STAT3 signaling pathway and downstream apoptotic molecules, some researchers revealed that GA had independent anticancer effects in non-small-cell lung cancer A549 cells and aided the anticancer effects of cisplatin. These findings might be used to justify more fundamental research and preclinical studies into GA’s anticancer properties and its auxiliary effects on cisplatin activity in non-small-cell lung cancer patients [38]. The results from the GCMS profiling depicted total 26 compounds. Some of these compounds such as palmitic acid, octadecadienoic acid, heptadecanoic acid, linoleic acid, cis vaccenic acid, and hexadecanoic acid have already been reported from bark extracts of other plant species, e.g., *Quercus leucotrichophora* [39]. Among the various compounds found, hexadeconic acid and vaccenic acid in particular have been investigated for their pharmacological potential, including anticancer [40,41] and anti-inflammatory properties [42]. Furthermore, the fraction’s activity might be attributed to the synergistic impact of these chemicals.

## 4. Materials and Methods

### 4.1. Chemicals and Reagents

Sigma Chemical Co. (St. Louis, MO, USA) provided the growth media for the cells, sulforhodamine B (SRB), crystal violet blue, Rhodamine 123, fetal bovine serum (FBS), and DCFDA (2,7-dichlorofluorescein diacetate). All additional chemicals were of analytical grade and obtained from standard sources.

### 4.2. Collection of Plant Material and Preparation of Extract and Fractions

A sufficient quantity of bark, leaves, and fruit of *C. dichotoma* was collected from district samba of UT J&K, India. To eliminate soil pollutants, the plant material was carefully rinsed under running tap water. The plant materials were chopped, dried in the shade, and powdered. The methanolic extract of the plant parts was obtained by impregnating the dried plant material in 95% methanol for 24 h with intermittent shaking. The mixture was filtered using filter paper and the residue was immersed in methanol for further 24 h. The filtrates were mixed and the mixture was concentrated to dryness under reduced pressure to produce a crude extract. Further fractionation of the crude (methanolic) extract was performed using n-hexane, chloroform, ethyl acetate and acetone solvents. All samples were concentrated and kept at −20 °C until further investigation.

### 4.3. Sample Preparation and Culturing of Cells

Stock solutions of 20 mg/mL were produced by dissolving the extract and subsequent fractions in DMSO. The human cancer cell lines A-549 (Lung), HCT-116 and SW-620 (Colon), MCF-7 and MDA-MB-231 (Breast), Mia PaCa-2 and Panc-1 (Pancreatic), SH-SY5Y, N2A, and U-251 (CNS) were acquired from the National Centre for Cell Science in Pune, India and the National Cancer Institute. These ten human cancer cell lines were developed and kept up with in RPMI-1640 media (A-549, SW-620, and MCF-7) and DMEM medium (HCT-116, MDA-MB-231, MIA PaCa-2, Panc-1, SH-SY5Y, N2A, and U-251).

### 4.4. SRB Assay for Cytotoxicity

The samples were examined for anticancer potential against ten distinct human cancer cell lines from five distinct origins: lung (A-549), colon (HCT-116, SW-620), breast (MCF-7, MDA-MB-231), pancreas (MIA PaCa-2 and Panc-1), and CNS (SHSY-5Y, U-251, and N2A). Cell suspensions of optimum cell density for A-549 (7500), HCT-116 (7000), SW-620 (13,000), MCF-7 (8000), MDA-MB-231 (8000), MIA PaCa-2 (10,000), Panc-1 (10,000), SH-SY5Y (15,000), U-251 (15,000), and N2A (10,000) were seeded in 96-well flat-bottom plates (NUNC). Cell suspension was added in an amount of 100 μL/well and the cells were allowed to proliferate for 24 h. Cells were subsequently grown for 48 h at 37 °C with test material (extract and fractions) including full growth medium (100 μL/well). Cell development was halted by carefully stacking 50 μL of chilled TCA in each well and incubating at 4 °C for one hour to fix the cells connected to the lower part of the wells. Each well’s liquid was then carefully pipetted out and disposed. The wells were cleaned and air-dried five times with distilled water. Each well received 100 μL of SRB and was incubated for 30 min at room temperature. Unbound SRB was removed from the cells by washing them five times with 1 percent *v*/*v* acetic acid. After drying at room temperature, tris buffer (100 μL) was added to each well to solubilize the color and plates were delicately swirled for few minutes on a mechanical stirrer. A microplate reader was used to measure the optical density at 540 nm [43].

### 4.5. Mechanistic Assays

#### 4.5.1. Evaluation of Cell Apoptosis

In a six-well plate, cells were planted at a density of 2 × 10^5^ cells/mL and incubated for 24 h. The chloroform fraction was added at concentrations of 50, 75, and 100 µg/mL for an additional 24 h. Following the incubation period, cells were splashed with PBS and fixed in 4 percent paraformaldehyde for 15 min at 4 °C. The fixed cells were washed again and stained with DAPI for 15 min in the dark. The stained cells were again splashed with PBS and analysed for nuclear morphological alterations using a fluorescent microscope (Olympus-1X53 magnification) [44].

#### 4.5.2. Estimation of Reactive Oxygen Species Production

Cells were seeded in a six-well plate (2 × 10^5^) and treated for 24 h with chloroform fraction (50, 75, and 100 µg/mL). 2 h before termination, H_2_O_2_ was introduced to the well-holding medium. The cells were then incubated for 30 min at 37 °C in the dark with 10 µM DCFDA (2, 7-dichlorofluorescein diacetate). The cells were washed with PBS before being inspected with a fluorescence microscope (Olympus-1X 53 magnifications). The fluorescence intensity was measured at 480/530 nm emission and excitation wavelengths [45].

#### 4.5.3. Colony Formation Assay

In a six-well plate, cells were seeded at a density of (2 × 10^5^) and cultured for 24 h. Following the incubation time, cells were treated with chloroform fraction at concentrations of 50, 75, and 100 µg/mL and incubated for another 24 h. The treated cells were then trypsinized, counted, and reseeded in a new six-well plate at 1000 cells per well. The plate was placed in an incubator for at least 6 cell divisions. The cells were rinsed with PBS after the medium was removed. After that, the cells were fixed in 1% formaldehyde for 20–25 min. The plate was kept at room temperature for 30 min after the fixed cells were stained with crystal violet dye. Following the incubation period, the crystal violet was removed, the plate was gently washed with water, and colonogenic survival was evaluated [46].

#### 4.5.4. Wound Healing Assay

Cells with density (3 × 10^5^)/well were seeded in a 6-well plate for 24 h. Then, using 200 µL tips at the top of the well, a horizontal scratch was formed on the cell monolayer and it was treated with chloroform fraction (50, 75 and 100 µg/mL) for the following 24 h. Cells were seen under the microscope at 0 h. After 24 h, the medium was aspirated, cleaned with PBS, and examined using a phase contrast microscope.

### 4.6. Analysis of Phytochemicals by HPLC and GCMS

In vitro cytotoxicity of extracts and fractions were tested against human cancer cell lines used in the present investigation. Based on the cytotoxicity results, chloroform fraction (ch fr) was picked for additional phytochemical portrayal by HPLC and GCMS.

Qualitative and quantitative analysis of phenols was performed by reverse-phase high-performance liquid chromatography (HPLC) using the method reported by Tarnawaski et al. [47]. A liquid chromatography system (Perkin Elmer, Boston, MA, USA) comprising of an HPLC pump, auto sampler, diode array detector (LC 200 D series), and Peltier Column oven (200 series) Perkin Elmer reverse phase column (150 × 4.6 mm, 5 μm C18) was used for analysis of phenols using 20 µL of samples. For analysis and data collection, total chrome workstation software (version 6.3.1.0504) was utilized. Mobile phase was comprised of acetic acid (2% *v*/*v*) in water and methanol (82:15, *v*/*v*) and a flow rate was adjusted at 1 mL/min. Analysis of phenolic acids chromatograms was performed at 280 nm on 30 min retention time frame. Standard phenolic compounds (gallic acid, ferulic acid, syringic acid, caffeic acid, vanillic acid, *p*-coumarin acid, *p*-hydroxy benzoic acid) at concentration of 1 mg/mL prepared in methanol were used. Phenolic compounds in samples were identified by comparing retention time of standard phenolic compounds with retention time of each phenolic compound.

Gas chromatography and mass spectroscopy were used to analyze the active fraction. The GC-MS 4000 (Varian, Atlanta, GA, USA) system was employed, along with an HP-5 MS agilent column (30 m × 0.25 mm i.d., 0.25 film thickness). The temperature of the injector was set at 280 degree Celsius. After holding the column at 50 degree Celsius for 5 min, the temperature was steadily raised by 3 degrees Celsius each minute up to 280 degree Celsius and then held constant at 280 degree Celsius for 7 min. Helium was used as the carrier gas at a constant flow rate of 1.0 mL/min. A sample solution of 0.2 L was put onto the column and examined. The MS scan settings comprised a 70 Ev electron impact ionization voltage, a mass range of 40–500 *m*/*z*, and the identification of the sample’s components was based on a comparison of their relative indexes.

### 4.7. Statistical Analysis

All experiments were conducted in triplicate and the findings were computed as mean ± standard deviation (SD). GraphPAD prism 5.0 was used to calculate IC_50_.

## 5. Conclusions

The assessment of anticancer activity of different parts of *C. dichotoma* against human cancer cell lines used in the study suggested that bark part of *C. dichotoma* might be a significant natural source for anticancer drugs. This is the first study to show that bark has anticancer properties against the A-549 cell line from lung origin. The chloroform fraction of bark has anticancer potential and causes cell death against A-549 human cancer cell line from lung origin via apoptosis induction mediated by excessive ROS production. Furthermore, HPLC and GCMS analyses revealed the presence of a number of bioactive molecules such as gallic acid, vanillic acid, ferulic acid, caffeic acid, palmitic acid, cis vaccenic acid, and others. We reported, for the first time, cis vaccenic acid from the bark through GC-MS. As a result of its promising efficacy, the fraction might be an essential cancer chemopreventive or chemotherapeutic drug for patients with lung carcinoma. To better comprehend their capacity to manage illnesses that have a significant impact on quality of life, more research into the separation of responsible anticancer components, as well as their mechanism of action, is required.

## 6. Patents

To best of our knowledge, this is the first study to show that the plant’s bark has anticancer properties against the A-549 human cancer cell line and induced chromatin condensation, confirmed cell death via ROS generation and significantly decreased colony formation in a dose dependent manner in A-549 cell line from lung origin. Furthermore, GC-MS investigations indicated the presence of the compound that was reported from the bark i.e., cis vaccenic acid, for the first time.

## Figures and Tables

**Table 1:** In vitro cytotoxicity IC_50_ of the chloroform fraction of bark and standard drug against human lung cancer cell line (A-549).

Cell Line	Chloroform Fraction of Bark IC50 (µg/mL)	Standard Drug (Paclitaxel) IC50 (nM)
A-549	37.978	4.8

**Table 2:** HPLC analysis of chloroform fraction of bark of *C. dichotoma*.

RT	Area (uV*sec)	Structure	Name
1.988	144,969.86	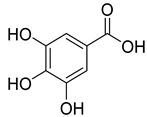	Gallic acid
4.842	2497.31	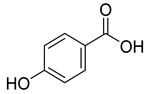	*p*-hydroxy benzoic acid
5.908	104.26	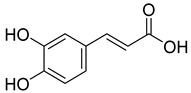	Caffeic acid
6.093	13,775.39	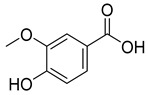	Vanillic acid
7.130	2724.56	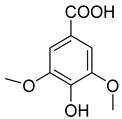	Syringic acid
10.909	275.75	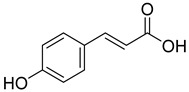	*p*-Counaric acid
14.218	472.87	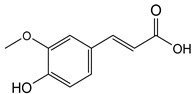	Ferulic acid

**Table 3:** Compounds found in chloroform fraction of *C. dichotoma* bark by GC-MS.

Compounds	Class of Compound	Area %	*m*/*z*	R. Time
1,3-Di-*tert*-butyl benzene	Phenyl propanes	0.68	175.10	14.527
4-Vinylguaiacol	Phenol	0.51	150.15	15.522
Diisobutyl phthalate	Ester	0.83	149.05	26.535
Methyl palmitate	Ester	1.29	74.00	27.365
Palmitic acid	Fatty acid	18.34	73	27.770
Benzylamine, 2-hydroxy-*N*,*N*-di[2-aminoethyl)amino]methyl)phenol	Phenol	1.73	71.05	27.935
16-Hydroxyhexadecanoic acid	Fatty acid	1.43	60.10	28.046
Methyl linoleate	Fatty acid	1.23	81.00	29.238
Methyl cis-9,10-epoxystearate	Ester	1.41	55.00	29.313
Octadecadienoic acid	Fatty acids	9.46	67.00	29.625
*cis*-Vaccenic acid	Fatty acids	28.81	55.00	29.681
1-Dimethyl(pentafluorophenyl)silyloxydodecane	NC	1.82	79.05	29.825
Palmitoleic acid	Fatty acids	12.54	73.00	29.915
1-(beta.-d-Ribofuranosyl)-4-difluormethoxy-uracil	Dihydropyridine	1.94	69.00	30.065
Methylallyl Trifluoroacetate	Allyl acyls	1.89	55.05	30.170
Difluoroacetic acid	Monocarboxylic acid	2.22	69.05	30.310
Linoleic acid trimethylsilyl ester	Ester	1.71	80.00	30.381
(2-phenyl-1,3-dioxolan-4-yl)methyl (z)-octadec-9-enoate	NC	0.85	73.00	30.440
Tridecyl 2-Methoxyacetate	Ester	0.70	57.00	31.389
6,9,12,15-Docosatetraenoic acid, methyl ester	Ester	0.57	69.10	32.196
Dotriacontane	Alkanes	1.58	57.00	32.656
(4-Allyloxy-3-methoxy-phenyl)-methanol	NC	0.58	97.15	32.756
1-Propyltridecyl 4-bromobutanoate	Ester	0.72	69.05	33.169
9-octylheptadecane	Alkanes	3.39	57.00	34.206
1-[(1-Ethylundecyl)oxy]-1-methylsilinane	Phenylpyrazoles	0.51	55.00	34.300
Dioctyl phthalate	Benzoic acid	2.66	148.95	34.778

NC—Not Classified.

## Data Availability

All data, tables and figures are original.

## Sample Availability

Samples of the compounds are available from the authors.
